# Scaling the respiratory metabolism to phosphorus relationship in plant seedlings

**DOI:** 10.1038/srep16377

**Published:** 2015-11-12

**Authors:** Zhi-Qiang Wang, Heng Huang, Jian-Ming Deng, Jian-Quan Liu

**Affiliations:** 1 MOE Key Laboratory for Bio-Resources and Eco-Environment, College of Life Sciences, Sichuan University, Chengdu 610065, Sichuan, People’s Republic of China; 2 State Key Laboratory of Grassland and Agro-Ecosystems, School of Life Sciences Lanzhou University, Lanzhou 730000, Gansu, People’s Republic of China

## Abstract

There are empirical indications of an isometric scaling relationship between plants’ respiratory metabolism rates and nitrogen contents. To test the hypothesis that there may be a similar relationship between plants’ respiratory metabolism and phosphorus contents we used data obtained from 150 laboratory and field-grown seedlings representing 30 herbaceous species and 20 woody deciduous species. Our results show that whole-plant respiration rates strongly scaled to the 0.81-power of the whole-plant phosphorus content, across wide ranges of growth conditions and functional classifications. Moreover, we also found a similar scaling exponent between whole-plant respiration rates and total nitrogen contents for the same set of samples. The similarities of the metabolic scaling relationships suggest that similar mechanisms may be involved in the transport and storage of phosphorus and nitrogen in plants.

Metabolic rates affect numerous (if not all) physiological and ecological processes^1^,^2^, via a general scaling relationship that can be described by the following power law equation:


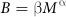


Here: *B* is a measure of metabolic rate, such as the respiration rate; *M* is body mass, β is a normalization constant and α is a scaling exponent. The value of α has stimulated vigorous debate^3^. West *et al*.^4^,^5^. proposed an integrated model of plant hydrodynamics, biomechanics and branching geometry, incorporating this equation, and determined α to be 0.75. Subsequently, several empirical and theoretical studies have demonstrated that the scaling exponent relating plant metabolic rates to body mass declines from nearly 1 for small seedlings and saplings to 0.75 for large plants^6^,^7^,^8^,^9^,^10^,^11^,^12^,^13^,^14^,^15^,^16^, due to shifts in physiological constraints on the allocation of plant biomass between photosynthetic and non-photosynthetic organs during ontogenetic progression.

As an essential component of key enzymes nitrogen is involved in crucial metabolic processes in plants and is tightly coupled with respiratory metabolism at multiple levels^17^,^18^,^19^,^20^,^21^. Furthermore, whole-plant respiration rates isometrically scale more consistently with total nitrogen content than with body mass^22^,^23^. Like nitrogen, phosphorus is a vital component of plants’ nucleic acids and many proteins, including enzymes involved in the respiratory release of energy contained in sugars and the regulation of numerous metabolic pathways^24^. Consequently, phosphorus is also considered to be a good predictor of metabolic rates in plants^25^,^26^,^27^, and it is required for all plant growth and development processes^26^,^28^,^29^. Thus, it seems reasonable to hypothesize that plants’ phosphorus contents are linked to their respiration rates through a scaling relationship similar to that observed for their nitrogen contents^12^,^22^,^23^,^30^. In the study presented here we tested this hypothesis through observations of 150 seedlings representing 30 herbaceous species and 20 deciduous woody species grown in either the laboratory or field. We measured phosphorus contents and respiration rates of the whole plants and their aboveground parts. In addition, to examine whether the hypothetical relationship (if present), is similar to that between nitrogen and respiration rates, we simultaneously measured the plants’ nitrogen contents.

## Results

**Figure 1:**
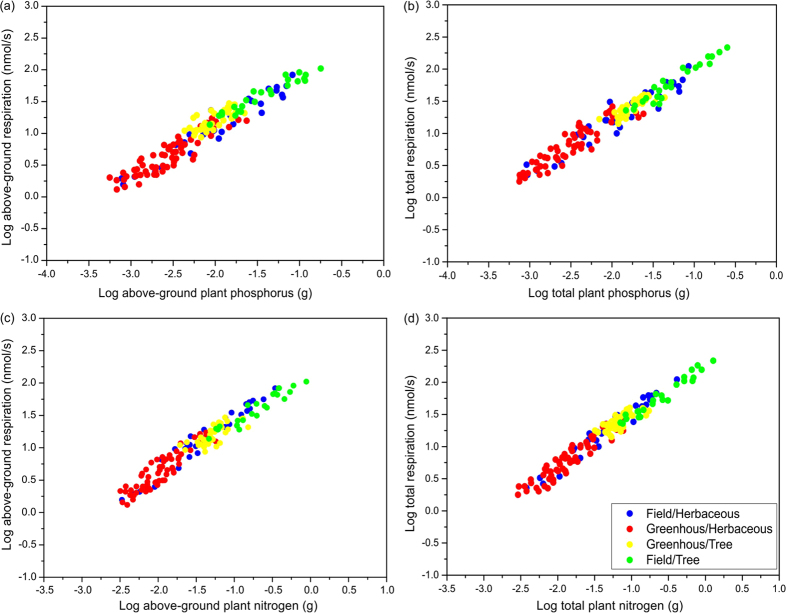
Log-log bivariate plots of aboveground respiration and total respiration rates (normalized to rates at 24 °C) in relation to phosphorus (a,b) and nitrogen (c,d) contents, determined from measurements of plants representing 30 herbaceous and 20 deciduous woody species grown in the field or a greenhouse, as indicated by the color-coding.

According to the pooled data for all seedlings of 50 plant species grown under greenhouse and field conditions (Table S1), the aboveground respiration rates scaled to the 0.81-power (95% CI =0.77–0.85, *r*^2^ = 0.923; *P* < 0.001) of the aboveground phosphorus content (Fig. 1a). Similar scaling relationships were also found for both functional groups (herbaceous and woody plants), under both greenhouse and field growth conditions (Table 1). The scaling exponent of whole-plant respiration rates to total phosphorus content (Fig. 1b) was also estimated to be 0.81 (95% CI = 0.77–0.83, *r*^2^ = 0.946; *P* < 0.001). Similar relationships between whole-plant respiration rates and phosphorus contents were also observed for both functional groups under both growth conditions (Table 1). Furthermore, the scaling relationships between nitrogen contents of both functional groups (either whole plants or their aboveground parts) under both growth conditions were very similar, with an estimated scaling exponent of ca. 0.82 (Fig. 1c,d; Table 2).

## Discussion

Our measurements of 150 small laboratory- and field-grown plants of 50 species (Table S1) indicate that there is a very strong scaling relationship between plants’ respiration rates and their phosphorus contents, with a scaling exponent of 0.81 for both whole plants and their aboveground parts that is not affected by the growth conditions. Furthermore, an extremely similar scaling exponent (0.82) was found between their respiration rates and nitrogen contents.

As elements that play numerous vital structural and functional roles in plants, phosphorus and nitrogen are likely to have similar uptake, transport, and allocation mechanisms for the following reasons. Both are mainly absorbed from the soil through root hairs^31^,^32^, and their further movements depend upon transport through cell membranes^33^. Both phosphorus and nitrogen are similarly unloaded into xylem vessels and transported upwards to the youngest leaves and other parts of the plant^33^,^34^,^35^. Their lateral movements in the vascular system are also similar^36^,^37^. For example, both can readily move from xylem to phloem. These observations regarding transport mechanisms suggest that both phosphorus andnitrogen contents may be constrained by the vascular distribution networks and thus have similar scaling relationships to respiration rates.

The scaling exponents for the respiration rate to nitrogen content relationships of plants we obtained differ from the 1.0-power obtained in a previous study^22^. However, in the cited study the biomass of the examined plants ranged from 0.01 to 1000 g while the biomass of our samples ranged from 0.1 to 200 g. Moreover, an estimated scaling exponent significantly exceeding 1.0 for the whole-plant respiration rate to total nitrogen content relationship was obtained in another study^12^, based on a set of samples with biomass ranging from 0.001 to 1 g. Thus, variations in the scaling exponent of respiration rates to nitrogen content may be at least partly due to variations in biomass ranges of the sampled plants. In addition, there may be considerable differences in scaling exponents between evergreen plants (which were not included in our study) and deciduous plants, because the former retain their leaves for many years and may accumulate increasing amounts of nitrogen and phosphorus, while leaves of the latter wither and are renewed annually. However, theoretical modelling indicates that the exponent of the relationship between respiration and biomass probably approaches 1.0 in small seedlings (body size < 1 g), but shifts to around 0.75 as plant biomass increases to 100 g^11^. The scaling exponent (0.82) of respiration to nitrogen estimated in our study based on a set of samples between 0.1 to 200 g seems to be consistent with such predictions. These findings suggest that a universal isometric relationship between whole-plant respiration rates and nitrogen content should be rejected^22^, but support the hypothesis that scaling relationships vary, depending on the biomasses of the examined plants^8^. In addition, the scaling relationship between respiration rates and phosphorus contents may vary similarly, as we discovered very similar allometric scaling relationships between respiration rates and both phosphorus and nitrogen.

## Materials and Methods

### Study sites

The study involved measurements of 150 plants representing 50 species of two functional groups (herbaceous and deciduous woody species; Table S1), some grown in a greenhouse at Lanzhou University’s Yuzhong Experimental Station, and others collected from the field at a site on Cuiying Mountain (35.946 N, 104.137 E; Gansu Province, China), also owned by Lanzhou University.

The material grown at the Experimental Station consisted of first-year seedlings of 20 herbaceous species and 2- to 4-year-old seedlings of 12 woody species grown in a white washed greenhouse (average temperature ≈ 22 °C, mean radiation ≈ 25% of full sunlight). At Cuiying Mountain (mean annual temperature ≈ 6.3 °C), first-year seedlings of 10 naturally regenerated herbaceous species and 2- to 3-year-old saplings of eight species of woody species were sampled. For further details see Table S1.

### Respiration measurements

Before measuring dark respiration rates, individual specimens were carefully dug up from the soil by hand and soil attached to the roots was washed off, to ensure that as few fine roots as possible were lost. Each entire plant was separated into aboveground parts (leaves plus stems) and belowground parts (roots), then placed in darkness for 30 min in preparation for measurement. A Li-8100 automated CO_2_ flux system (LI-COR, Nebraska, USA) was used to record the dark respiration rates in a customized chamber (3.5 L volume)^16^. Three replicate measurements were taken from three individual plants per species, each measurement lasting 5 min. The ambient temperature was recorded during all the respiration measurements. To account for temperature effects on dark respiration rates, measured rates were adjusted to corresponding rates at a standardized temperature (24 °C) using a previously published temperature-dependent Q_10_ model^19^. Whole-plant respiration rates were estimated by summing the aboveground and root respiration rates.

### Phosphorus and nitrogen measurements

All of the samples of aboveground parts and roots were dried at 120 °C for 30 min, followed by 65 °C for 72 h then weighed to determine their dry weights. Dried tissue samples, including a mixture of leaves and stems (aboveground), or roots (belowground), were powdered using a mortar and pestle. The nitrogen contents of portions of the powdered samples were then analyzed using a 2400II CHNS/O Element Analyzer (Perkin-Elmer, Boston, MA, USA), with furnace temperature set at 950 °C for combustion then reduced to 640 °C. Phosphorus contents of portions of the powdered above- and below-ground tissue samples were also analyzed, using the molybdate/ascorbic acid method after H_2_SO_4_–H_2_O_2_ digestion^38^. The nitrogen and phosphorus contents of the aboveground parts and roots of the sampled plants were then calculated by multiplying their measured nitrogen and phosphorus concentrations (g g^−1^) and dry biomasses (g). The total plant nitrogen and phosphorus contents were calculated by simply summing the aboveground (leaves and stems) and belowground (roots) contents.

### Statistical analysis

All data were log_10_-transformed to allow expression of the power function in the form of a linear regression equation, which was used to estimate parameters for each variable and confidence intervals for the parameters^39^,^40^. Type II (reduced major axis) regression models were used to determine scaling exponents (α) and normalization constants (β) from the log_10_-transformed data using SMATR Version 2.0^41^,^42^.

## Additional Information

**How to cite this article**: Wang, Z.-Q. *et al*. Scaling the respiratory metabolism to phosphorus relationship in plant seedlings. *Sci. Rep*. **5**, 16377; doi: 10.1038/srep16377 (2015).

## Supplementary Material

Supplementary Table S1

## Figures and Tables

**Table 1:** Scaling relationships between respiration rates and phosphorus contents of the sampled plants (for all cases, *P* < 0.001).

Material	*n*	intercept	95% CI	exponent	95% CI	*r*^2^
Aboveground						
Field/herbaceous	30	2.71	2.54,2.87	0.81	0.73,0.89	0.935
Greenhouse/herbaceous	60	2.70	2.46,2.93	0.80	0.71,0.89	0.819
Greenhouse/woody	36	2.86	2.48,3.24	0.82	0.63,1.01	0.577
Field/woody	24	2.46	2.36,2.57	0.62	0.55,0.69	0.932
All	150	2.76	2.67,2.84	0.81	0.77,0.85	0.923
Whole plant						
Field/herbaceous	30	2.79	2.61,2.97	0.80	0.71,0.89	0.917
Greenhouse/herbaceous	60	2.95	2.72,3.18	0.85	0.76,0.94	0.842
Greenhouse/woody	36	2.84	2.56,3.13	0.85	0.66,0.98	0.694
Field/woody	24	2.87	2.75,3.00	0.86	0.76,0.96	0.935
All	150	2.81	2.74,2.87	0.81	0.77,0.83	0.946

**Table 2:** Scaling relationships between respiration rates and nitrogen contents of the sampled plants (for all cases, *P* < 0.001).

Material	*n*	intercept	95% CI	exponent	95% CI	*r*^2^
Aboveground						
Field/herbaceous	30	2.39	2.27,2.50	0.89	0.82,0.97	0.953
Greenhouse/herbaceous	60	2.54	2.34,2.74	0.96	0.86,1.07	0.839
Greenhouse/woody	36	2.20	1.96,2.43	0.78	0.60,0.96	0.559
Field/woody	24	2.12	2.04,2.20	0.74	0.64,0.84	0.905
All	150	2.24	2.19,2.29	0.82	0.78,0.85	0.940
Whole plant						
Field/herbaceous	30	2.45	2.35,2.54	0.89	0.82,0.95	0.963
Greenhouse/herbaceous	60	2.44	2.29,2.59	0.88	0.80,0.96	0.882
Greenhouse/woody	36	2.26	2.06,2.47	0.75	0.58,0.92	0.583
Field/woody	24	2.25	2.20,2.30	0.80	0.73,0.87	0.957
All	150	2.33	2.29,2.37	0.82	0.79,0.85	0.956
